# The association between medically unexplained physical symptoms and health care use over two years and the influence of depressive and anxiety disorders and personality traits: a longitudinal study

**DOI:** 10.1186/s12913-016-1332-7

**Published:** 2016-03-22

**Authors:** Madelon den Boeft, Jos W. R. Twisk, Berend Terluin, Brenda W. J. H. Penninx, Harm W. J. van Marwijk, Mattijs E. Numans, Johannes C. van der Wouden, Henriette E. van der Horst

**Affiliations:** Department of General Practice and Elderly Care Medicine, EMGO Institute for Health and Care Research, VU University Medical Center, van der Boechorststraat 7, 1081 BT Amsterdam, The Netherlands; Department of Epidemiology and Biostatistics, VU University Medical Center, van der Boechorststraat 7, 1081 BT Amsterdam, The Netherlands; Department of Psychiatry and EMGO Institute for Health and Care Research, VU University Medical Center, A.J. Ernststraat 1187, 1081 HL Amsterdam, The Netherlands; Department of Public Health and Primary Care, Leiden University Medical Center, Albinusdreef 2, 2333 ZA Leiden, The Netherlands

**Keywords:** Medically unexplained physical symptoms, Somatisation, Health care use, Depressive disorders, Anxiety disorders, Personality traits

## Abstract

**Background:**

Medically unexplained physical symptoms (MUPS) are highly prevalent and are associated with frequent health care use (HCU). MUPS frequently co-occur with psychiatric disorders. With this study we examined the longitudinal association between MUPS and HCU over 2 years and the influence of depressive and anxiety disorders and personality traits on this association.

**Methods:**

We analysed follow-up data from 2045 to 2981 participants from the Netherlands Study of Depression and Anxiety (NESDA), a multisite cohort study. The study population included participants with a current depressive and/or anxiety disorder, participants with a lifetime risk and/or subthreshold symptoms for depressive and/or anxiety disorders and healthy controls. HCU, measured with the Trimbos and iMTA questionnaire on Costs associated with Psychiatric illness (TIC-P), was operationalized as the number of used medical services and the number of associated contacts. MUPS were measured with the Four Dimensional Symptoms Questionnaire, depressive and anxiety disorders with the Composite International Diagnostic Interview and personality traits with the NEO Five-Factory Inventory. Measurements were taken at baseline, 1 and 2 years follow-up. We used generalized estimating equations (GEE), using HCU at all three measurements as (multivariate) outcome. GEE also takes into account the dependency of observations within participants.

**Results:**

MUPS were positively associated with HCU over 2 years (medical services: RR 1.020, 95 % CI 1.017–1.022; contacts: RR 1.037, 95 % CI 1.030–1.044). Neuroticism and depression had the strongest influence on the associations. After adjustment for these factors, the associations between MUPS and HCU weakened, but remained significant (services: RR 1.011, 95 % CI 1.008–1.014; contacts: RR 1.023, 95 % CI 1.015–1.032).

**Conclusions:**

Our results show that MUPS were positively associated with HCU over 2 years, even after adjusting for depressive and anxiety disorders and personality traits.

**Electronic supplementary material:**

The online version of this article (doi:10.1186/s12913-016-1332-7) contains supplementary material, which is available to authorized users.

## Background

Medically unexplained physical symptoms (MUPS), physical symptoms that cannot be explained or not sufficiently explained by an underlying medical condition after adequate examination, are highly prevalent in all health care settings [1–5]. MUPS represent a broad spectrum of symptoms in varying degrees of severity, ranging from acute, mild MUPS to severe and chronic MUPS [6, 7]. It is known that patients with MUPS have a high health care use (HCU) leading to high costs [8–10]. Therefore, MUPS put a burden not only on patients and physicians, but also on society in a time when health care costs are steadily rising.

This high HCU is regularly attributed to patients pressurizing their general practitioner (GP) for a somatic treatment for their symptoms. However, several studies suggest that most patients do not request somatic interventions but want support and acknowledgement of the reality of their symptoms, but instead receive interventions initiated by the GP [11, 12].

Several studies showed that patients with MUPS use disproportionally large amounts of mostly somatic health care services and not particularly mental health care services [13, 14]. Barsky et al. for instance found that primary care patients with MUPS had approximately twice the outpatient and inpatient HCU and twice the annual medical costs compared to non-MUPS patients [8]. Studies that have been performed on this topic used different methodological approaches. Many of them used a retrospective design [8, 14, 15], only included patients from primary care [3, 8, 13, 16] or only included patients with severe MUPS [3, 17]. As far as we know, only one recent study used a prospective design with extended follow-up and included a large sample of participants from the general population [18].

It is also known that MUPS frequently co-occur with depressive and/or anxiety disorders [19–21]. This is of great clinical relevance as this ‘cosyndromality’ leads to more disability, impairment and high HCU [8, 18, 22]. The same applies to some personality traits such as neuroticism. Although in literature most research has been performed on personality disorders that are associated with MUPS [23], Noyes et al. suggested that certain personality traits co-occurring with MUPS could lead to increased care seeking behaviour [24]. These findings raise the question what the independent association of MUPS with HCU is and to what extent personality traits and depressive and anxiety disorders add to this association.

For our study, we used data from the Netherlands Study of Depression and Anxiety, a large naturalistic multisite longitudinal cohort. Data on MUPS, HCU, depressive and anxiety disorders and personality traits were all collected over time from a large sample of participants from several health care settings. Therefore, this cohort is ideally suited to investigate the following research questions: What is the association between MUPS and HCU over 2 years? And, to what extent is the association between MUPS and HCU influenced by depressive and/or anxiety disorders and specific personality traits?

## Methods

### Design, setting and study sample

The Netherlands Study of Depression and Anxiety (NESDA) aims to describe the long-term course and consequences of depressive and anxiety disorders and to examine its predictors. A detailed description of its rationale and design has been published elsewhere [25]. In summary, the study sample consisted of 2981 participants (age 18–65) with current depressive and/or anxiety disorders, participants with a lifetime risk or subtreshold depressive and/or anxiety symptoms and healthy controls. Recruitment took place across primary care practices (*n* = 1610), outpatient secondary mental health care institutions (*n* = 807) and the general population (*n* = 564). Exclusion criteria were not being fluent in the Dutch language and a primary diagnosis of a psychotic, obsessive compulsive, bipolar or severe substance abuse disorder. Baseline data were collected between 2004 and 2007. Assessments, including written questionnaires and interviews, were repeated after 1, 2, 4 and 6 years. Non-response among participants was not significantly related to mental health status, but slightly higher among younger and male respondents. The research protocol was approved centrally by the ethical review board of VU University medical center. Subsequently it was approved by the local ethical review boards of Leiden University Medical Center and University Medical Center Groningen. The study was performed in accordance with the ethical standards of the Declaration of Helsinki. All participants provided written informed consent.

For the present study, we used data of all participants who completed the questionnaire used for our study, and used the measurements at baseline (T0), one (T1) and 2 years (T2) of follow-up. Baseline measurements were obtained from 2981 participants. At T1 and T2, 2045 (68.6 %) and 2395 (80.3 %) participants had a follow-up assessment, respectively.

### Health care use (HCU), the outcome

HCU was measured with the Trimbos and iMTA questionnaire on costs associated with psychiatric illness (TIC-P) [26]. The TIC-P is a widely used, feasible and reliable questionnaire on health care consumption and productivity losses for patients with mental health disorders. For this study we focused on the first part of the TIC-P, consisting of dichotomous questions on relevant medical services, followed by a question on the consumption volume (number of contacts) in the past 6 months; e.g. ‘did you consult with a family physician? No/Yes, namely … times’. We counted the number of medical services used (range 0–14) and additionally categorized these into three subgroups: mental health care services (primary care psychologists, social workers/social psychiatric nurses, secondary mental health care institutions, centers for drugs or alcohol, self-help groups and private psychiatrists/psychotherapists); somatic health care services (family physicians, medical specialists and hospital admissions); and miscellaneous health care services (homecare, complementary alternative professionals, occupational health physicians, physiotherapists). Participants completed the TIC-P at T0, T1 and T2.

### Medically unexplained physical symptoms (MUPS), the determinant

MUPS were measured with the somatisation scale of the validated Four Dimensional Symptoms Questionnaire (4DSQ) [27]. The self-report 4DSQ has been developed to measure distress, depression, anxiety and somatisation as separate dimensions. The somatisation scale comprises 16 items including physical symptoms that often remain medically unexplained (e.g. dizziness and abdominal pain). The scale highly correlates with instruments used in other countries measuring MUPS; 0.82 in case of the SCL-90 [27, 28] and 0.84 in case of the PHQ-15 [29, 30]. In the present sample Cronbach’s alpha of the 4DSQ somatisation scale was 0.92, 0.89 and 0.97 at the three measurements, respectively. The items on the somatisation scale are scored on a 5-point Likert scale: “no”, “sometimes”, “regularly”, “often”, and “very often or constantly”. In order to arrive at scale scores, the responses were recoded as 0 for “no”, 1 for “sometimes” and 2 for “regularly”, “often” and “very often or constant” and summated, resulting in a score ranging from 0 to 32. Additionally, in order to facilitate clinical use and to overcome the fact that there is no linear relation between MUPS and HCU, we repeated the analyses with a dichotomized scale using 11 points as a cut-off score, since a score of 11 or higher is considered to indicate MUPS [27]. Participants completed the 4DSQ at T0, T1 and T2.

### Depressive and anxiety disorders

The presence of depressive and anxiety disorders was assessed with the validated Composite International Diagnostic Interview (CIDI, WHO 2.1) at T0 and T2. Trained research staff interviewed all participants. Depressive disorders included major depressive disorder and dysthymia. Anxiety disorders included generalized anxiety disorder, panic disorder with or without agoraphobia, social phobia and/or agoraphobia without panic disorder. We only took into account diagnoses established during the previous six months at both assessments.

### Personality traits

Personality traits were measured with the NEO Five Factor Inventory (NEO-FFI) at T0 and T2. The NEO-FFI measures the five most important personality domains in adults: neuroticism, extraversion, openness, agreeableness and conscientiousness. Each domain is measured with 12 items, using a five-point Likert response format (sum score: range 12–60). More detailed information about the contents, validity and reliability of the NEO FFI has been published elsewhere [31–33].

### Sociodemographic variables and chronic diseases

Based on previous studies, we considered the following sociodemographic variables as possible confounders: gender, age, level of education, marital status and the number of chronic diseases [34, 35]. The level of education was derived from the standard classification of education from Statistics Netherlands [36] and categorized into three groups (basic, intermediate, high). Marital status divided participants in those being married/living with a partner and those living alone. Participants were asked if they had one or more diseases from the following chronic diseases categories: respiratory, cardiometabolic, musculoskeletal, digestive, neurological, endocrine and cancer. We only considered and summated the diseases if participants were currently treated with medication and/or under specialist control. All variables were assessed at T0.

### Statistical analysis

Descriptive statistics are presented as mean with standard deviation for normally distributed continuous data, median and inter-quartile range for skewed continuous variables and as numbers and percentages for dichotomous and categorical variables.

**Fig. 1:**
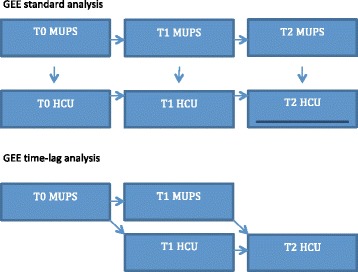
Analysis models T0: baseline; T1: 1 year follow-up; T2: 2 years follow-up. MUPS: medically unexplained physical symptoms. HCU: health care use

Generalized estimating equations (GEE) with an exchangeable correlation structure were used to assess the relationship between MUPS and HCU longitudinally (Fig. 1). We used GEE because it takes into account the dependency of repeated observations within the participants and because it is capable of analysing non-complete longitudinal data. As the total number of consulted medical services showed a Poisson distribution, we used Poisson GEE analysis to assess its association with MUPS. For the total number of contacts, we used negative binomial GEE analysis because the Poisson distribution was skewed to the right (a Poisson distribution with overdispersion). The effect sizes of both the Poisson and the negative binomial GEE analyses are expressed as rate ratios (RRs). This RR represents the association between MUPS and HCU on average over time and reflects both a within and between subjects interpretation [37]. Besides crude analyses, we adjusted the relationships for the sociodemographic variables, and additionally we examined the influence of depressive and anxiety disorders and personality traits on the association between MUPS and HCU. The influence is expressed as the percentage decrease in the regression coefficient as a result of including each separate variable. As depressive and anxiety disorders and personality traits were only measured at T0 and T2, these last analyses were based on these two measurements only. We repeated the crude and adjusted analyses for each of the three medical resource subgroups. Furthermore, as we performed our analyses in a population with an oversampling of depressive and anxiety disorders, we analysed whether the effect between MUPS and HCU was modified by depressive and/or disorders by adding an interaction term to the model. Finally, we carried out the same set of analyses with a time lag model in order to assess if MUPS at a certain point in time was related to HCU one year later (Fig. 1). We used all observations in our analyses.

All regression coefficients were considered to be statistically significant when the *p*-value was below 0.05. All statistical analyses were performed in SPSS 20.0 for Windows.

The reporting of this manucripted adhered to the STROBE guidelines (Additional file 1).

## Results

Table 1 shows the descriptive information of all variables used in this study. At baseline, the mean age was 42 years and 66 % were women. The mean score for MUPS was 10 at T0, 8.6 at T1 and 8.1 at T2. When using the clinical cut-off point of 11, 42 % had MUPS at T0, 27 % at T1 and 25 % at T2.

**Table 1:** Sample characteristics

	Baseline	One year follow-up	Two year follow-up
*Socio-demographics*			
Females, number (%)	1979 (66.4)		
Age in years, mean (SD)	41.9 (13.1)		
Level of education, number (%)			
Basic	199 (6.7)		
Intermediate	1736 (58.2)		
High	1046 (35.1)		
Number of chronic diseases, mean (SD)	0.6 (0.9)		
Married or with partner, number (%)	2066 (69.3)		
*MUPS (4DSQ somatisation scale)*			
Total score (0–32), mean (SD)	10.0 (7.1)	8.6 (6.7)	8.1 (6.3)
Dichotomized: number with MUPS (≥11) (%)	1237 (42.0)	806 (27.0)	741 (24.9)
*Health Care Use (TIC-P)*			
Total number of medical services (0–12), mean (SD)	2.4 (1.5)	2.5 (1.6)	2.9 (1.8)
Mental health care services	0.6 (0.8)	0.5 (0.8)	0.6 (0.9)
Somatic health care services	1.2 (0.7)	1.4 (0.8)	1.4 (0.9)
Miscellaneous health care services	0.6 (0.8)	0.7 (0.8)	0.8 (0.9)
Total number of contacts with medical services, median (IQR)	7.0 (14.0)	9.0 (19.0)	12.0 (28.0)
Mental health care contacts	0.0 (5.0)	0.0 (5.0)	0.0 (8.0)
Somatic health care contacts	3.0 (4.0)	4.0 (4.0)	3.0 (5.0)
Miscellaneous health care contacts	0.0 (5.0)	0.0 (6.0)	2.0 (14.0)
*Depressive or anxiety disorders (CIDI), number (%)*			
Depressive disorders	1158 (38.8)	-	626 (21.0)
Anxiety disorders	1305 (43.8)	-	711 (23.9).
*Personality score (NEO-FFI; 12–60), mean (SD)*			
Neuroticism	36.3 (9.4)	-	33.5 (9.0)
Extraversion	36.9 (7.4)	-	37.8 (7.2)
Openness	38.2 (6.0)	-	36.8 (5.3)
Agreeableness	43.8 (5.3)	-	44.5 (5.2)
Conscientiousness	41.7 (6.5)		42.3 (6.2)

*MUPS* medically unexplained physical symptoms, *4DSQ* four dimensional symptom questionnaire, *TIC-P* Trimbos and iMTA questionnaire on costs associated with psychiatric illness, *IQR* interquartile range (25th to 75th percentile), *CIDI* composite international diagnostic interview, *NEO-FFI* NEO five-factor inventory

### The longitudinal association between MUPS and HCU

MUPS were significantly associated with the total number of consulted medical professionals and the total number of associated contacts, respectively, on average over time in both the crude and adjusted GEE analyses (Table 2). To illustrate the interpretation of the results: the estimated adjusted RR of 1.02 found for MUPS in relation to the total number of medical resources can be interpreted as follows: for every unit increase in the 4DSQ, a 2 % increase in the number of medical services is observed, within and between participants. The small difference between the crude and adjusted analyses was mainly driven by the number of chronic diseases. MUPS defined with the dichotomized 4DSQ showed results in the same direction (services: RR 1.35; 95 % CI 1.31–1.40; contacts: RR 1.41; 95 % CI 1.28–1.55, not in table). Outcome defined as the three categories of medical services also showed comparable results (Table 2). The strongest association was found for both the number of mental health care services used and the number of contacts with these services. Table 3 shows the influence of depressive and anxiety disorders and personality traits on the association of HCU with MUPS. For both HCU outcomes, neuroticism had the strongest influence, followed by depressive disorders. When taking neuroticism and depressive disorders together, the magnitude of the regression coefficient decreased by 48 % (services) and 44 % (contacts). Despite the contribution of these mental health characteristics, HCU remained significantly associated with MUPS. Adding anxiety disorders and other personality traits did not further affect the association. Also for the dichotomized 4DSQ score, neuroticism and depressive disorders together showed the strongest influence (decrease in regression coefficients of 56 and 59 %, respectively) and HCU remained significantly associated with MUPS. When we examined whether the association between MUPS and HCU was modified by depressive and/or anxiety disorders, we found a significant inverse interaction effect (*p* < 0.001), meaning that the association between MUPS and HCU (both services and contacts) was weaker for patients with depressive and anxiety disorders (data not shown).

**Table 2:** MUPS and HCU over time: GEE standard analyses

	Crude RR (95 % CI)	Adjusted RR (95 % CI)
MUPS and total number of medical services	1.022 (1.020–1.024) *	1.020 (1.017–1.022) *
Somatic services	1.013 (1.011–1.015) *	1.009 (1.006–1.011) *
Mental health care services	1.035 (1.030–1.039) *	1.035 (1.030–1.040) *
Miscellaneous health care services	1.027 (1.023–1.031) *	1.026 (1.021–1.030) *
MUPS and total number of contacts	1.045 (1.040–1.052) *	1.037 (1.030–1.044) *
Somatic contacts	1.044 (1.040–1.048) *	1.031 (1.027–1.035) *
Mental health care contacts	1.046 (1.035–1.058) *	1.044 (1.031–1.057) *
Miscellaneous health care contacts	1.042 (1.034–1.050) *	1.029 (1.020–1.038) *

* All RRs including 95 % CIs were significant with *p*-values below 0.001. The adjusted RRs were adjusted for the sociodemographic variables and chronic diseases. All measurements include T0, T1 and T2. Mental health care services: primary care psychologists, social workers/social psychiatric nurses, secondary mental health care institutions, centers for drugs or alcohol, self-help groups and private psychiatrists/psychotherapists. Somatic health care services: family physicians, medical specialists and hospital admissions. Miscellaneous health care services: home care, complementary professionals, occupational health physicians and physiotherapists

**Table 3:** MUPS and HCU over time, adjusted for depression, anxiety and personality: GEE standard analyses

	MUPS and number of medical services (RR; 95 % CI)	Percentage decrease in regression coefficient	MUPS and number of contacts (RR; 95 % CI)	Percentage decrease in regression coefficient
Adjusted RR ^a^	1.021 (1.019–1.024)*		1.041 (1.034–1.048)*	
Depressive disorders	1.015 (1.013–1.018)*	29	1.031 (1.024–1.039)*	24
Anxiety disorders	1.018 (1.015–1.020)*	14	1.034 (1.026–1.042)*	17
Neuroticism	1.013 (1.011–1.016)*	38	1.027 (1.018–1.036)*	34
Extraversion	1.018 (1.016–1.021)*	14	1.036 (1.028–1.044)*	12
Openness	1.021 (1.019–1.024)*	0	1.041 (1.034–1.048)*	0
Agreeableness	1.021 (1.019–1.024)*	0	1.039 (1.031–1.047)*	5
Conscientiousness	1.020 (1.017–1.022)*	0	1.037 (1.030–1.045)*	10
Neuroticism & Depressive disorders	1.011 (1.008–1.014)*	48	1.023 (1.015–1.032)*	44

* All RRs were significant with *p*-values below 0.001. ^a^ All analyses were based on measurements at T0 and T2, as depression, anxiety and personality were not measured at T1. Therefore, a new rate ratio was calculated, adjusted for sociodemographic variables and chronic diseases

### MUPS related to HCU one year later

Table 4 shows the results of the time-lag analyses. When comparing the results of the time-lag analyses with the standard analyses, we found comparable RRs for the number of medical services, but slightly higher RRs for the number of contacts in the time-lag analyses. For the dichotomized 4DSQ score, results were in the same direction (services: RR 1.19; 95 % CI 1.15–1.22; contacts: RR 1.64; 95 % CI 1.48–1.83). For the influence of depressive and anxiety disorders and personality traits on the association between MUPS and HCU, we found the same pattern with neuroticism as the strongest influencing variable, followed by depressive disorder and again even stronger when taken together (data not shown). MUPS was still associated with HCU over a longer period of time, as reflected in the time lag analyses (data not shown).

**Table 4:** MUPS related to HCU 1 year later: GEE time-lag analyses

	Crude RR (95 % CI)	Adjusted RR (95 % CI)
MUPS and number of medical services	1.021 (1.018–1.023)*	1.018 (1.015–1.020)*
MUPS and number of contacts	1.060 (1.053–1.067)*	1.051 (1.043–1.058)*

*All RRs were significant with *p*-values below 0.001. The adjusted RRs were adjusted for the sociodemographic variables and chronic diseases. MUPS were measured at T0 and T1. HCU was measured at T1 and T2

## Discussion

In the present study we found a positive association between MUPS and HCU over 2 years taking into account all measurements, both for the number of medical services as well as the associated contacts. After adjusting for depressive and anxiety disorders and personality traits, the associations weakened, especially due to depressive disorders and neuroticism, but remained statistically significant.

### Comparison with literature

Our findings on the positive association between MUPS and HCU are in accordance with previous studies, irrespective of the differences in methodology [8, 10, 13, 17, 18, 22]. However, when we examined the three categories of medical resources, we found the strongest association between MUPS and mental HCU, in contrast to some other studies [8, 13, 18]. Fink et al. concluded in their study among patients with somatoform disorders that these patients used more non-psychiatric health care facilities than patients without somatoform disorders [13]. Also Barsky et al. found large amounts of medical, but not mental, health care use among their somatising patients [8]. Their findings support the assumption that patients with MUPS attribute their complaints to their physical symptoms, thereby seeking somatic health care instead of mental health care. The difference with our findings could be explained by the difference in setting (primary care only versus primary care, mental health care and general population).

We found that depressive disorders and neuroticism had the strongest influence on the association between MUPS and HCU over time. As far as we know, no research has been published on the comorbidity of MUPS with psychiatric disorders and personality traits with regard to HCU, especially not in a longitudinal design. De Waal et al. found that somatoform disorders and depressive disorders were almost equally associated with HCU, but that the undifferentiated somatoform disorder had an independent effect after adjusting for psychiatric disorders [22]. Noyes et al. found that MUPS were associated with specific personality traits as neuroticism and that this led to increased care seeking behaviour, which is in accordance with our findings [24]. However, in contrast with our findings, Carlier et al. found no association between somatoform disorders and specific personality traits in their cross-sectional study [38]. As an explanation for this result they argued that their somatoform disorders patients were mostly highly educated and married, indicating a stable personal life.

### Generalizability of the results

One should realize that we examined the relation between MUPS and HCU in a sample with predominantly depressive and/or anxiety disorders. This may impede the interpretation and generalizability of the results. Therefore, we investigated possible effect modification between MUPS and depressive disorders and between MUPS and anxiety disorders. These analyses showed that within the population without these disorders the relationship between MUPS and HCU was stronger than in the population with these disorders. Based on those analyses, we believe that the observed relations may also hold for the general population.

### Strengths and limitations

A main strength of our study was that we used the NESDA cohort to answer our research questions. By using this cohort, longitudinal data over 2 years from a large sample of participants were available, recruited from both primary care, secondary mental health care and the general population. Also, we adjusted all analyses for chronic somatic diseases. Furthermore, we used structured diagnostic interviews and not only self-report questionnaires.

However, our findings should be interpreted in the light of several limitations. First, the NESDA cohort study used the 4DSQ somatisation scale to measure MUPS. As with all existing MUPS questionnaires, it lacks judgement of a clinician to verify that symptoms are really unexplained [39]. However, the 4DSQ highly correlates with the PHQ-15 and SCL-90, questionnaires that are widely used to measure MUPS [27], and may be considered as an adequate proxy measure for MUPS. Second, HCU was measured over the past 6 months, while MUPS were measured over the past week, leading to incongruence. However, we do not believe that the results were affected much by this. We found a correlation coefficient of 0.72 of MUPS over the 2 years (data not shown), indicating a quite stable pattern of MUPS over time. Also, our time-lag analyses showed results that were similar to those of the standard analyses, with even a slightly higher effect of MUPS on HCU for the number of contacts. Third, as HCU was asked over the past 6 months, the risk of recall bias exists. However, the direction of this bias is unclear. Other studies have used electronic medical records to assess HCU, but these can also be incomplete [40]. Fourth, we have no information on the actual reasons for health care use and have only assessed the quantity and not the appropriateness of provided health care. Also we did not control for the use of psychopharmacological therapy which could have influenced the results, nor can we determine whether MUPS were the primary problems or symptoms from a psychiatric disorder. Fifth, only the baseline sociodemographic variables and number of chronic diseases were included as covariates in the analyses, although several of these covariates may have changed over the course of 2 years. However, we do not believe that the results of our adjusted analyses would be different because the influence of these covariates was only marginal. Finally, based on our study we cannot infer causality. Therefore it might at least theoretically be possible that an increase in HCU is leading to more MUPS.

### Implications for clinical practice and future research

As we found that MUPS are independently associated with HCU, attention should be paid to early identification and adequate treatment of MUPS in clinical practice. Also, physicians should be aware of signs of depression and anxiety and personality traits as they have an influence on the association between MUPS and HCU.

A possible explanation for prolonged high HCU is that care for patients with MUPS is often fragmented, as diagnostics and treatments are carried out by different health care providers. To reduce high and possibly inadequate health care for these patients, the issue of adequacy and fragmentation of health care patterns should be further examined, also in relation to the patient’s quality of life. Guidelines for MUPS across disciplines and different health care settings may be instrumental in this examination [41, 42].

Also, it would be interesting to examine the role of health anxiety as a predictor of HCU in a longitudinal design. As we found neuroticism to be a predictor, it is possible that health anxiety could play an important role as well as these are often related.

## Conclusions

Our study showed that MUPS are positively associated with HCU over time, even after adjustment for depressive and anxiety disorders and personality traits. This suggests that good MUPS management is important. Further research is needed to investigate the adequacy of health care use patterns and the association with the patient’s quality of life.

## Additional file

Additional file 1: STROBE Statement—checklist of items that should be included in reports of observational studies. (DOC 86 kb)

## Abbreviations

MUPS: medically unexplained physical symptoms
HCU: health care use
NESDA: Netherlands study of depression and anxiety
TIC-P: Trimbos and iMTA questionnaire on costs associated with psychiatric illness
4DSQ: four dimensional symptoms questionnaire
CIDI: composite international diagnostic interview
WHO: World Health Organization
NEO-FFI: NEO five factor inventory
GEE: generalized estimating equations
RR: rate ratio
